# Integration of Mixed Reality Technology Into a Global Neurosurgery Bootcamp

**DOI:** 10.7759/cureus.63888

**Published:** 2024-07-05

**Authors:** Jack P Rock, Lonni Schultz, Robert Dempsey, Jonathan Cohen

**Affiliations:** 1 Neurological Surgery, Henry Ford Health System, Detroit, USA; 2 Neurological Surgery, University of Wisconsin School of Medicine and Public Health, Madison, USA; 3 Neurosurgery, Hoth Intelligence, Philadelphia, USA

**Keywords:** medical education, mixed reality, augmented reality, global health, global neurosurgery

## Abstract

International bootcamps are important for providing access to advanced education and training to physicians around the world. In countries where resources are scarce, the opportunity to be exposed to advanced training and the latest technologies is limited. We set out to evaluate the educational value of integrating augmented reality (AR) into the curriculum of a global neurosurgery bootcamp. AR was integrated into this year’s neurosurgical bootcamp in Hanoi, Vietnam, organized by the Foundation for International Education in Neurological Surgery (FIENS). Participants had not experienced this technology before a surgical adjunct. A study was conducted to evaluate how AR impacts the surgical approach to a cranial tumor for boot camp participants with limited neurosurgical experience. Without the use of AR, the majority of participants (66%) chose the incorrect surgical approach to a frontal tumor. However, after using AR to visualize the lesion in 3D, all participants chose the correct surgical approach. Additionally, participants were more precise when planning with AR as the distance from the skull insertion point to the tumor was significantly shorter with AR than without AR. This study demonstrated the potential of AR to improve the education and enhance the experience trainees have at international bootcamps. Importantly, it is our hope that industry involvement in these global initiatives continues to grow as it is critical for trainees in developing countries to be exposed to common as well as emerging medical technologies.

## Introduction

In the realm of neurosurgery, access to advanced education and training stands as a crucial cornerstone for enhancing surgical expertise and improving patient outcomes. However, in many countries, the scarcity of resources and the lack of specialized training facilities present significant barriers to delivering comprehensive neurosurgical education. Augmented reality (AR) emerges as a transformative solution, offering a promising avenue to bridge these gaps and revolutionize neurosurgery education in resource-constrained regions [1,2]. AR, with its immersive capabilities, has transcended traditional learning methods, offering unparalleled opportunities for neurosurgeons, medical students, and healthcare professionals to enhance their understanding, skills, and proficiency in this intricate discipline [1]. The integration of AR in neurosurgery education holds immense potential to address the education that exists in other countries [3]. By leveraging AR technology, medical practitioners and aspiring neurosurgeons can gain access to immersive, interactive, and high-quality educational resources [4].

Here, we describe the integration of AR into the curriculum of a neurosurgical bootcamp in Hanoi, Vietnam. These bootcamps, initiated by the Society of Neurosurgery in 2014 have continued annually internationally since 2017 [5-7]. For this year’s bootcamp, the organizers sought to introduce novel AR technology into the curriculum as this technology has gained considerable traction in the field of neurosurgery over the past few years. In this report, we conducted a study utilizing AR to educate bootcamp participants about cranial approaches to a brain tumor. The AR system was used to provide an immersive experience for visualizing cranial anatomy from a clinical case. Subsequently, participants used the AR system to plan a cranial trajectory, and the impact of AR on their chosen surgical approach was evaluated. This exploration aims to shed light on the possibilities and the influence that AR may hold in shaping the landscape of neurosurgical education on a global scale. While this represents an initial step towards bringing immersive technology to global neurosurgical education, it is our hope and expectation that there will be a continued trend of integrating advanced technologies into the curriculum of international bootcamps in developing countries.

## Materials and methods

Participants

The bootcamp was attended by neurosurgical trainees from Vietnam as well as various neurosurgical faculty from around the world (Table 1). The experience levels of bootcamp participants ranged from intern level to senior resident/fellow. The exercise described in this study was supervised by a representative from Hoth Intelligence Inc., Philadelphia, and a senior neurosurgical attending.

**Table 1:** Participant Institution Affiliations

General Hospital Nghe An Province
Can Tho University of Medicine and Pharmacy
National Cancer Institute
Saint Paul General Hospital
Thanh Nhan Hospital
Can Tho Central General Hospital
E Hospital
University of Danang
General Hospital of Thanh Hoa Province
Gia Dinh People's Hospital
Hanoi Medical University
Hospital 105 Son Tay
Hospital 175
Huu Nghi Hospital
K Tan Trieu Hospital
Kon Tum Provincial General Hospital
Long An General Hospital
Ho Chi Minh University of Medicine and Pharmacy
Military Hospital 103
Military Hospital 175
Ba Ria Hospital
General Hospital of Nghe An Province
Neurosurgery Department - Phu Tho Provincial General Hospital
Neurosurgery, Bac Giang Provincial General Hospital
Quang Ngai Provincial General Hospital
Resident doctor at Ho Chi Minh City University of Medicine and Pharmacy
Thai Nguyen Central Hospital
Tien Giang Provincial General Hospital
Trieu An General Hospital
Vietnam-Germany Hospital
Xuyen A General Hospital
Yen Bai Provincial General Hospital

Generating a 3D model

**Figure 1:**
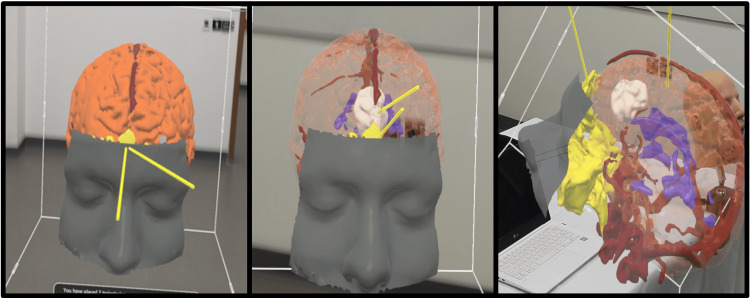
Users' Experience Through an Augmented Reality Headset Participants’ view of the 3D model through the headset: brain, sinuses, vasculature, ventricles, tumor.

A 3D model of a frontal tumor clinical case was generated from a T1 magnetic resonance imaging (Figure 1). The 3D model consisted of the face, brain, sinus, tumor, vasculature, and ventricles. The model was segmented using a 3D slicer. The segmentation and resulting 3D model were confirmed by the surgeon who performed that case. Files were exported and loaded onto the Microsoft Hololens 2 headset [8-10].

AR system

An AR visualization system was used to view and interact with the 3D-reconstructed anatomic model. The system operates out of the Microsoft Hololens 2 headset, which is an untethered optical see-through AR display that superimposes virtual content onto the surrounding “real world.” During this workshop, the participants were able to visualize the 3D model, manipulate the model with their hands, and visualize the 3D anatomy from different perspectives by moving around the 3D model while wearing the headset (Figure 1).

Study design

**Figure 2:**
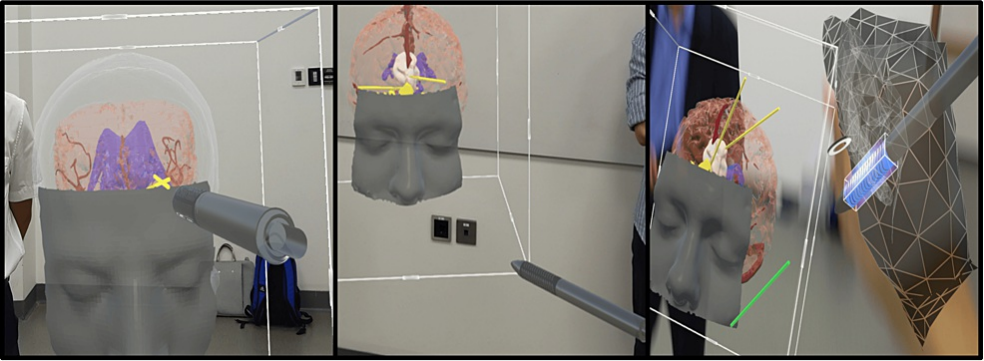
Virtual Pointer Tool Used to Place Trajectories on the 3D Model

Participants all received a didactic introduction to AG and its current applications in the field of neurosurgery. After the didactic session, participants were shown a T1 MRI of a frontal tumor case. For this case, clinically, the location of the craniotomy was chosen based on the shortest distance to the tumor. Participants were able to visualize the location of the tumor in axial, sagittal, and coronal planes on a computer. After studying the MRI, participants put on the AR headset and visualized the 3D model. Initially, the participants were only able to see the face and brain layer of the 3D model and were not able to see the location of the intracranial tumor. The AR system has built-in planning tools such as virtual marking pens that allow users to place and superimpose trajectories in their field of view (Figure 2). The participants were asked to mark and plan a trajectory towards the tumor based on where they perceived the tumor to be from analyzing the MRI scan alone. After placing an initial trajectory, the brain on the 3D model was made transparent, revealing the 3D tumor on the 3D model. With the ability to visualize the tumor on the 3D model, the participants were asked again to place a 2nd trajectory line of their surgical approach to the tumor. At the conclusion of the exercise, the model contained two trajectory lines - a trajectory line towards the tumor based on visualizing the tumor location from the MRI alone and the trajectory towards the tumor placed while visualizing the tumor on the 3D model.

Data analysis

After participants placed trajectories, a picture through the headset was captured. Additionally, the 3D coordinates of the center of the tumor, as well as the trajectory lines, were stored. 3D spatial coordinates of all virtual objects are stored by the headset. The frontal approach was the correct approach for this particular case, based on it being the shortest path to the tumor. As such, we measured the distance from the insertion point of each trajectory - the location in which the virtual trajectory line intersects with the outer surface of the brain on the 3D model - the center of the tumor. Therefore, for the purposes of this study, we were able to assess whether and how much participants’ surgical approach to the tumor was changed when planning based on the MRI alone versus planning the approach when visualizing the tumor in the 3D model.

Statistical analysis

We performed a statistical comparison between distance-to-tumor for trajectory lines placed based on MRI only and AR guidance. The distances were compared via Students’ t-test. McNemar’s test was done to compare the MRI-only and AR guidance approaches.

## Results

Each participant planned two trajectory approaches towards the tumor. A summary of the insertion approaches for each trajectory is shown in Table 2. Prior to using AR, only 33.3% of participants chose the correct surgical approach to the tumor. When visualizing the tumor location in AR, 100% of participants chose the correct frontal approach. As such, once able to visualize the 3D tumor (as opposed to the 2D tumor on the DICOM), the correct frontal approach was taken by all participants. To quantity quantify how far the participants altered their surgical approach after visualizing the tumor in 3D, we measured the distance between the initial insertion point-defined as the location in which the trajectory line intersects with the brain on the 3D model-with the insertion point placed while visualizing the tumor in 3D. After using AR to place the second trajectory, participants moved the location of their insertion location by an average of 4.6 ± 2.4 cm.

**Table 2:** Cranial Approaches Taken With and Without Augmented Reality Visualization

Participant	Non-AR	AR Assisted
1	Frontotemporal	Frontal
2	Frontal	Frontal
3	Frontal	Frontal
4	Temporal	Frontal
5	Frontotemporal	Fontal
6	Endonasal	Frontal
7	Frontal	Frontal
8	Frontal	Frontal
9	Frontal	Frontal
10	Temporal	Frontal
11	Temporal	Frontal
12	Frontotemporal	Frontal
13	Occipital	Frontal
14	Frontotemporal	Frontal
15	Temporal	Frontal
16	Frontal	Frontal
17	Frontotemporal	Frontal
18	Temporal	Frontal

**Figure 3:**
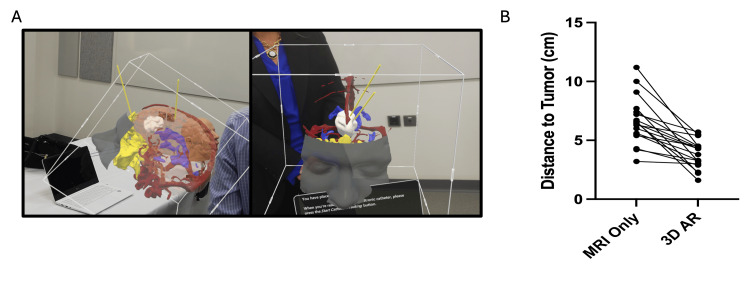
Trajectory Placement Using the AR System A) Representative examples of trajectory placements by the bootcamp participants as viewed through the headset. (B) Quantification of the distance to tumor viewing the tumor on the 2D magnetic resonance image (MRI) versus the 3D augmented reality (AR) model.

The distance from the insertion point to the center of the tumor was measured based on spatial coordinates saved by the AR headset. When placing trajectories based on the interpretation of the 2D MRI alone, the average distance from the brain insertion point to the tumor was 6.67 ± 1.99 cm. The distance of the insertion point to the tumor for the second trajectory (planned when inserting the 3D tumor) was significantly shorter than the first trajectory with an average distance of 3.81 ± 1.17 cm (Figure 3). As such, upon visualizing the tumor on the 3D model, the participants identified a shorter path to the tumor.

## Discussion

Bootcamps are an effective means of supporting neurosurgical faculty and young trainees in North America and in developing countries. Based on the Society of Neurological Surgeons (SNS) resident education model developed in 2010, bootcamps in the United States have consisted of a two-day training course, focusing on the basics of neurosurgical care. A curriculum is set in place during which lectures are given on day one, followed by hands-on skill stations on day two [5,7]. Residents can establish a baseline of neurosurgical competencies in procedures that they will need to perform during their residencies and staff may participate, observe, or teach. Under the auspices of the Foundation for International Education in Neurosurgery (FIENS), pioneering international bootcamps in developing countries have acted as a model for exposing trainees to a wide range of neurosurgical topics to help improve patient care in their region. Given the significant variability in neurosurgical training in each country, bootcamps may assist with the development of a standardized curriculum for young training programs [11-13]. An often overlooked aspect of training initiatives in developing countries is the exposure to emerging technologies. Typically, there is a delay in the timing with which neurosurgical trainees in developing countries gain exposure and experience with new technologies entering the field [14,15]. As such, we sought to introduce an emerging neurosurgical technology - AG - into the workshop experience at an international bootcamp.

At the Hanoi bootcamp, various levels of experience among the residents were noted. Participants were given the opportunity to learn about and use AG during a presurgical planning exercise. For nearly all participants, this was their first exposure to this class of technologies. The exercise allowed participants to leverage the advantages of 3D AR visualization to plan a surgical approach to a cranial lesion. At the AG workstation, it was noted that, regardless of the level of experience, all residents, when guided by AG, devised the correct plan for a surgical approach as compared to using 2D DICOM visualization alone. These conclusions were drawn based on various participants changing their trajectory to the correct frontal approach after using AR as well as determining a shorter path to the tumor. This observation supports the idea that, by using three-dimensional AG guidance, even less experienced residents can perform at the level of their more senior co-residents, thereby facilitating and expediting the learning process. For this case, given the tumors' location, the correct surgical approach was chosen based on the shortest path to the lesion. However, we recognize that the shortest path is not always the correct path. Therefore, future studies are warranted to explore the impact of AR for challenging cases in which additional factors such as surrounding anatomy and proximity to eloquent brain regions must be considered when choosing a surgical approach.

Rapid advancement and adoption of cutting-edge technologies is a core component of neurosurgery. In developing countries, trainee exposure to current technologies can be limited. As such, an active and collaborative effort from neurosurgeons, institutions, and industry is required in order to facilitate greater access to emerging technologies. It is our hope that this sets a precedent for future workshops and bootcamps to encourage the continued introduction of new technologies. These initiatives promote greater engagement and participation of workshop attendees and improve the overall learning experience.

## Conclusions

The integration of AR technology in neurosurgical bootcamps represents a leap forward in medical education and training. AR has proven to be a valuable tool in enhancing the learning experience for aspiring neurosurgeons by offering immersive, interactive, and hands-on training opportunities. Especially in developing countries, where access to emerging technologies is limited, the introduction of this technology and others like it provides a new and engaging platform for neurosurgical education.

## References

[1] Dey A, Billinghurst M, Lindeman RW, Swan JE 2nd (2018). A systematic review of 10 years of augmented reality usability studies: 2005 to 2014. Front Robot AI.

[2] Lee K (2005). Augmented reality in education and training. Tech Trends.

[3] Cannizzaro D, Zaed I, Safa A (2022). Augmented reality in neurosurgery, state of art and future projections. A systematic review. Front Surg.

[4] Meola A, Cutolo F, Carbone M, Cagnazzo F, Ferrari M, Ferrari V (2017). Augmented reality in neurosurgery: a systematic review. Neurosurg Rev.

[5] Ament JD, Kim T, Gold-Markel J (2017). Planning and executing the neurosurgery boot camp: the Bolivia experience. World Neurosurg.

[6] Selden NR, Anderson VC, McCartney S, Origitano TC, Burchiel KJ, Barbaro NM (2013). Society of Neurological Surgeons boot camp courses: knowledge retention and relevance of hands-on learning after 6 months of postgraduate year 1 training. J Neurosurg.

[7] Lepard JR, Corley J, Sankey EW (2020). Training neurosurgeons in Myanmar and surrounding countries: the resident perspective. World Neurosurg.

[8] Sánchez-Margallo JA, Miguel CP de, Anzules RAF, Sánchez-Margallo FM (2021). Application of mixed reality in medical training and surgical planning focused on minimally invasive surgery. Front Virtual Real.

[9] Palumbo A (2022). Microsoft HoloLens 2 in medical and healthcare context: state of the art and future prospects. Sensors (Basel).

[10] Gsaxner C, Li J, Pepe A, Jin Y, Kleesiek J, Schmalstieg D, Egger J (2023). The HoloLens in medicine: a systematic review and taxonomy. Med Image Anal.

[11] Neylan CJ, Nelson EF, Dumon KR (2017). Medical School surgical boot camps: a systematic review. J Surg Educ.

[12] Zhang J, Zilundu PL, Zhang W, Yu G, Li S, Zhou L, Guo G (2022). The use of a surgical boot camp combining anatomical education and surgical simulation for internship preparedness among senior medical students. BMC Med Educ.

[13] Schmitt F, Eyssartier E, Sarfati-Lebreton M, Rony L, Boucher S, Riquin E, Martin L (2022). Preparatory surgical bootcamp: an effective form of training with a positive impact on self-confidence and procedural skills of the residents. Surg Prac Sci.

[14] Ramsey KM, Weijer C (2007). Ethics of surgical training in developing countries. World J Surg.

[15] Ozgediz D, Roayaie K, Debas H, Schecter W, Farmer D (2005). Surgery in developing countries: essential training in residency. Arch Surg.

